# The Rise of Non-typhoidal Salmonella Infections in India: Causes, Symptoms, and Prevention

**DOI:** 10.7759/cureus.46699

**Published:** 2023-10-08

**Authors:** Radhika A Dudhane, Nandkishor J Bankar, Yogendra P Shelke, Ankit K Badge

**Affiliations:** 1 Microbiology, Datta Meghe Medical College, Datta Meghe Institute of Higher Education and Research, Wardha, IND; 2 Microbiology, Jawaharlal Nehru Medical College, Datta Meghe Institute of Higher Education and Research, Wardha, IND; 3 Microbiology, Bhaktshreshtha Kamalakarpant Laxmanrao Walwalkar Rural Medical College, Ratnagiri, IND

**Keywords:** prevention, extended spectrum beta-lactamase, antimicrobial resistance, non-typhoidal, non-typhoidal salmonella infections

## Abstract

Non-typhoidal Salmonella infections (NTS) are a growing concern in India, posing a significant health risk to the population. These infections are becoming more common at worrisome rates, primarily because of inadequate surveillance. Salmonella non-typhoidal causes severe gastroenteritis and can even cause invasive infections, such as bacteremia, and focal infections, such as meningitis and septic arthritis, and is acquired through contaminated food and water sources. From moderate to severe, the symptoms might vary. Certain serovars exhibit a stronger propensity for specific syndromes, with serious infections more commonly observed in vulnerable populations. Consuming contaminated food, using inadequate sanitation procedures while handling meat from animals slaughtered, and contaminated water supplies are some of the causes of these diseases. Proper food and water treatment, better sanitary facilities, public awareness campaigns, and adherence to food safety laws are all part of prevention measures. The issue of antimicrobial resistance further emphasizes the necessity for prudent antibiotic usage. The Indian government has put in place programs including public awareness campaigns, better sanitary facilities, and stricter food safety laws. In the future, efforts should, however, concentrate on improving laws, boosting hygienic practices, and funding the development of new medicines and vaccines. These actions will lessen the burden of NTS infections in India by assisting in their prevention and management. This review aims to understand the reasons for this growing tendency, which is essential for creating efficient control and prevention strategies.

## Introduction and background

Non-typhoidal Salmonella (NTS) infections, the most prevalent form of infection caused by Salmonella, have grown to be a significant public health concern in India [1,2]. Due to its effects on morbidity, mortality, and healthcare expenditures, this increase has alarmed medical professionals, legislators, and the general public. NTS infections are either transmitted through a zoonotic reservoir or there may be person-to-person transmission. Salmonella transmission has been linked to contaminated food and water sources, including incorrectly handled poultry, eggs, meat, and vegetables [3]. Inadequate food safety standards and oversight, poor sanitation and hygiene practices, and others all contribute to the spread of the disease [4]. NTS infections can manifest as mild to severe symptoms and frequently lead to gastroenteritis [5]. Typical symptoms include fever, nausea, vomiting, diarrhea, and abdominal pain. This infection may spread outside of the digestive tract systemically by either invading the bloodstream or propagating into the lymphatic system, contributing to remote abscess formation of the pathogen in various organs, which require rapid and correct diagnosis for optimal care and to stop the infection from spreading further [6]. The prevention of NTS infections is essential. Contamination can be considerably decreased by bringing into practice effective food safety procedures, including correct cooking methods, safe food handling, and thorough washing of fruits and vegetables [7]. Other modes of prevention include improved sewage systems, access to clean water, and educating people on improved sanitation practices like the availability of household latrines and safe waste management, and hygiene practices like handwashing with soap and water [8,9]. The objective of the study is to offer valuable information that would help in understanding and addressing NTS infections, a growing public health problem.

## Review

Methods

The literature search was conducted through a review of electronic databases like PubMed, Google Scholar, and Scopus using appropriate keywords like "Non-Typhoidal Salmonella," "Salmonella infections," "causes," "symptoms," and "prevention."

**Figure 1:**
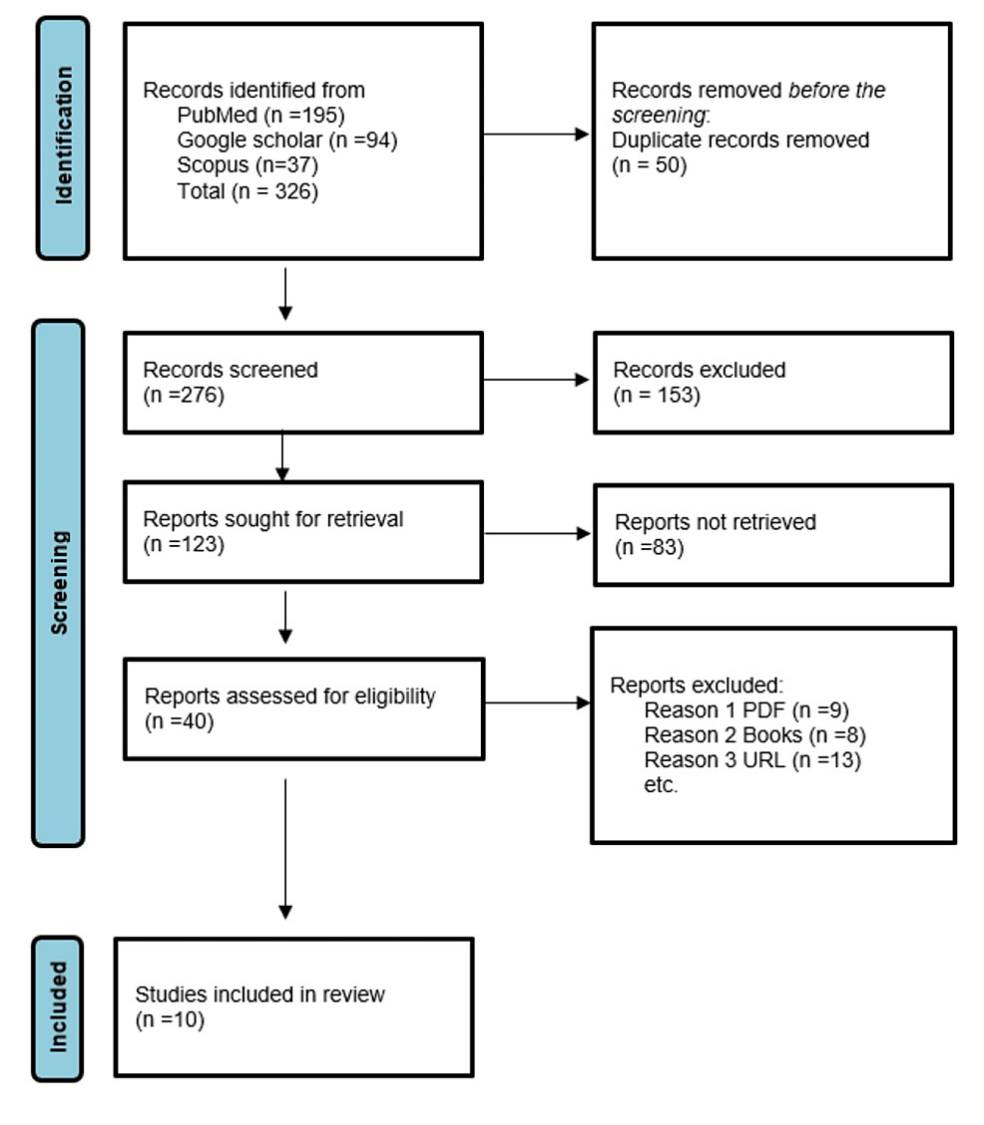
PRISMA n, number of studies; PRISMA, Preferred Reporting Items for Systematic Reviews and Meta-Analyses

Based on relevancy, research design, and publication date, filter publications were selected. Articles were included till 2023 to guarantee that the material is current. Research that was not specific to NTS was excluded. Data was sorted for analysis. The final review included a total of 10 articles from the years 2015 to 2023 (Figure 1).

Articles included in the review show a comparative analysis of various isolates of NTS infections (Table 1). 

**Table 1:** Studies included in the review NTS, non-typhoidal Salmonella; AMR, antimicrobial resistance; ESBL, extended-spectrum beta-lactamase; ERIC, enterobacterial repetitive intergenic consensus; PCR, polymerase chain reaction; PFGE, pulsed-field gel electrophoresis; ST313 and UGA14, types of Salmonella Typhimurium

Sr. No	Authors	Insights	Methods used	Practical implications	Results	Summarized abstract
1	Jain P et al. [10]	NTS infections are important causes of acute gastroenteritis in children in Kolkata, India, with high rates of antimicrobial resistance.	Rectal swabs collected from children with acute gastroenteritis. Standard procedures for identification of NTS.	Continuous monitoring of antimicrobial resistance profiles is recommended. Control the spread of resistant organisms.	1% Salmonella isolates recovered from 9957 samples. S. Worthington (33%) was the predominant serovar.	Study on NTS isolates from children in Kolkata. India Identified serovars, antimicrobial resistance, and molecular subtypes.
2	Pragasam AK et al. [11]	This study reports a 5% prevalence of ceftriaxone-resistant NTS infections in South India over the past nine years.	Minimum inhibitory concentration determined by E-test and broth microdilution method. Screening of AMR gene markers for beta-lactamases and ESBLs.	Rising incidence of ceftriaxone resistance in NTS. Monitoring antimicrobial resistance in this species is crucial.	5% prevalence of ceftriaxone resistance in NTS beta-lactamases mediate ceftriaxone resistance.	Ceftriaxone-resistant NTS is increasing in South India. Resistance is mainly mediated by ESBL
3	Patra S et al. [12]	The study found that invasive NTS infections are a significant cause of morbidity and mortality in southern India.	Retrospective study on culture-confirmed cases of bacteremia. Diagnosis of infection done by blood culture.	Invasive NTS disease is a severe infection with high mortality rate in India. Continued surveillance is necessary to monitor for multidrug-resistant strains.	Incidence of invasive NTS was 0.1 per 100 blood cultures. Majority of invasive NTS patients were male (77.5%).	Study on invasive NTS disease in southern India. Describes clinical presentation, antimicrobial susceptibility, and outcome.
4	Sudhaharan S et al. [13]	This study analyzed the extraintestinal infections caused by non-typical Salmonella in a care center in India.	A retrospective study of the relevant demographic, clinical, and laboratory data.	NTS infections are an emerging infection. Early diagnosis and management are crucial for patients.	Predominant age group: 20-30 years. Salmonella typhimurium is the most common NTS isolated.	Study analyzed extraintestinal infections caused by NTS. Salmonella typhimurium was the predominant strain isolated.
5	Subburaju N et al. [14]	There is a paucity of data on NTS infections in India.	Demographic, clinical, and laboratory data analyzed for NTS bacteremia.	Infants younger than 12 weeks with febrile and toxic features should be treated. Antibiotics may prolong the carrier state.	13 children with NTS bacteremia were analyzed. No deaths and nil complications were reported.	NTS primarily transmitted from animals. NTS bacteremia occurs in 5% of cases.
6	Kumar Y et al. [15]	Salmonella Newport is a non-typhoidal serovar of Salmonella that has been reported as a major cause of foodborne infections in India.	Standard methods used for antibiogram data generation.	Efficient surveillance programs should be implemented. Steps toward formulation and execution should be taken.	Salmonella Newport isolates were received from eight states and one union territory in India. S. Newport isolates exhibited resistance to most drugs.	Salmonella Newport is a major cause of foodborne infections.
7	S. Saravanan et al. [16]	The study found a higher incidence of NTS contamination in poultry tissue and animal protein sources in India.	Cultural and biochemical methods for identification. Multiplex PCR, allele-specific PCR, ERIC PCR, PFGE.	Higher incidence of NTS contamination in poultry products. Further investigation needed to evaluate risk factors and control measures.	21 out of 1215 samples tested positive for NTS. All Salmonella isolates were resistant to oxytetracycline.	NTS contamination found in poultry tissue and products. Salmonella Typhimurium and Salmonella Enteritidis identified as major serovars.
8	Oommen S et al. [17]	Data on NTS in India is limited, but this study found 15 cases of NTS in central Kerala.	Retrospective study on blood and stool culture samples. Standard bacteriological methods including serotyping with specific antisera.	Understanding prevalence and sensitivity patterns of NTS. Useful for epidemiological and treatment purposes.	15 cases of NTS were isolated. Three isolates were associated with bacteremia.	Prevalence and epidemiology of NTS in central Kerala. Most common isolates: Salmonella Typhimurium and Salmonella Enteritidis
9	Gajurel D et al. [18]	The given text does not provide information about NTS in India.	Retrospective study. Antimicrobial susceptibility testing performed on isolates.	Need to reevaluate fluoroquinolone therapy in Nepal. Introduce feasible alternatives to curb treatment failures.	High frequency of nalidixic acid-resistance in Kathmandu. Over 50% ciprofloxacin and ofloxacin-resistance in both serovar.	Drug-resistant Salmonella species is endemic in Asia. High frequency of nalidixic acid-resistance in Kathmandu.
10	Kubicek-Sutherland JZ et al. [19]	The text does not provide any information about NTS in India.	Genotypic and phenotypic analyses were performed. Biology phenotypic arrays and growth on brilliant green agar were used.	Advancing bio-surveillance efforts to control invasive NTS. Informing countermeasures against antimicrobial-resistant NTS.	Nine out of 11 isolates were identified as S. Typhimurium ST313. UGA14 isolate displayed novel plasmid, pseudogene, and resistance features.	Comparative analysis of invasive NTS isolates from Siaya, Kenya. Identification of novel plasmid, pseudogene, and resistance features in Salmonella Typhimurium isolate UGA14.

Out of the ten articles included, eight are from India and the other one each from Nepal and Kenya. While one study focuses on the study of NTS isolates from children as a cause of acute gastroenteritis, one study shows the prevalence of ceftriaxone-resistant NTS infections in Southern India [10,11]. Two studies are based on the inspection of invasive NTS disease [12,19]. Isolates of Salmonella Typhimurium were identified as major serovars in three studies [13,16,17]. Another study reported Salmonella Newport to be a major cause of foodborne infections [15]. A high frequency of nalidixic acid-resistance NTS in Kathmandu was observed in a study [18]. In a study, 5% of the cases show the occurrence of NTS bacteremia [14]. 

Salmonella, a pathogenic bacteria in the *Enterobacteriaceae* family, causes enteric diseases in humans and is of global concern. Transmission of NTS poses threats to public health but still, it has been overlooked in the Indian population due to poor surveillance [20]. These infections are brought on by a particular set of bacteria that spread through contaminated food and water sources and cause gastroenteritis involving diarrhea, fever, stomach cramps, and vomiting as well as other health problems and range in intensity from mild to severe [21]. Certain serovars have a great propensity to develop a certain condition and in severe cases can even lead to death. Thus it is essential to understand the causes, symptoms, and prevention methods [22]. Generally, infants, people over the age of 50, and individuals with immunocompromised conditions are susceptible to severe infections [23].

Causes of non-typhoidal salmonella infections

The primary mode of transmission of NTS infections is through the feco-oral route [24,25]. Salmonella Newport, a non-typhoidal serovar, has been identified as a significant contributor to outbreaks of disease such as diarrhea, ileocecal lymphadenitis, chest wall abscess, pyosalpinx, osteomyelitis, endocarditis, meningitis, splenic abscess, septicemia, and bacteremia that are brought on by the ingestion of contaminated food products [15]. The bacteria can survive in the intestines of animals and birds, and when this meat or eggs is consumed by humans, the bacteria can be transmitted to the human body. It is necessary to identify the severity of the problem to consider the zoonotic potential of food-borne bacteria and their propensity to create toxins [26]. Additionally, fruits and vegetables that are grown using contaminated water or fertilizers can also carry the bacteria. Since it employs eco-agricultural practices that are allegedly ecologically benign and provides goods that may be free of pesticide residues, organic agriculture has been practiced and gaining the attention of the food manufacturing sector. However, using animal manure in organic agricultural practices might increase the danger of infection by enteric pathogenic bacteria, which may then offer adverse health effects [27,28].

NTS infection transmitted through meat products develops when animals go through slaughtering and raw meat is handled and transported in an unhygienic process, which is due to poor sanitation infrastructure [20,29]. NTS spreading via the environment and water resources is through the micro- and macro-fauna surviving through improperly treated sewage having distinct physiological tolerance levels [30,31]. Various outbreaks reveal an unambiguous association of NTS infection and direct or indirect contact with infected animals or their surroundings. Infected animals carry Salmonella species without presenting any symptoms, making them potential key reservoirs for NTS infections. Another infection pathway includes insufficient hand cleaning and contact with sick pets and the bacteria cause infection by colonizing the digestive tract after being taken in infectious amounts [32,33].

Common symptoms of non-typhoidal salmonella infections 

The symptoms of NTS infections typically appear within 12 to 72 hours of exposure to the bacteria and their severity depends on a range of factors, including the age and health of the infected person, as well as the strain of the bacteria responsible for the infection [20]. In immunocompetent people, non-typhoidal salmonellae mostly trigger self-limiting enterocolitis, which is characterized by nausea, vomiting, extensive watery diarrhea, and abdominal discomfort [34]. Following diarrhea, these bacteria can survive in the gastrointestinal system, the risk of which is increased due to antibiotic medication [35]. Without accompanying diarrhea, this is known as primary NTS bacteremia and its symptoms include abdominal cramps, fever, and vomiting [36,37]. NTS invasive infections can be difficult to identify and address early due to clinical similarities with febrile infections and its severe complications include osteomyelitis, lung infections, sepsis, dehydration, septic arthritis, and even death [38]. Furthermore, intestinal perforation is an extremely rare and potentially fatal complication of severe NTS infection [39]. Prompt diagnosis and treatment of NTS invasive infections is crucial to prevent the progression of the disease and minimize the risk of complications. In severe cases, hospitalization may be required for intravenous antibiotic therapy and supportive care. Patients with underlying health conditions, such as compromised immune systems, are particularly vulnerable to NTS infections and should take extra precautions to avoid exposure. Public health measures, such as proper food handling and hygiene practices, can also play a crucial role in preventing the spread of NTS infections in the community [33].

Potential risk factors and transmission

In at-risk populations, individuals who are immunosuppressed due to steroid usage, HIV infection, chronic renal, cancer, diabetes, or sickle-cell disease, as well as elderly and infants, primary NTS bacteremia without concomitant diarrhea can develop [30]. Patients with structural abnormalities, such as biliary or urinary tract abnormalities, valvular heart disease, atherosclerosis or aneurysms, bone abnormalities, or prostheses, may also develop metastatic infection [40-42]. Salmonellosis is a zoonosis containing a large animal reservoir. The primary and most common animal reservoirs are pigs, cows, turkeys, and chickens. These organisms can be found in dozens of other domestic and wild animals. Animal products are the primary means of transmission for salmonella due to the organism's ability to survive in raw meats and other animal products [43-45].

A total of 999 suspected NTS isolates from various locations in India were received at NSEC between 2016 and 2018 for serovar confirmation. A total of 539 (53.95%) out of 999 isolates were serologically and biochemically verified to be NTS; the remaining isolates have been identified to be non-Salmonella (n=362, 36.23%) and typhoidal Salmonellae (n=98, 9.8%), and they were excluded from the research. Most NTS isolates (n=313, 58.07%) were collected from South region of India, and other regions contributed between 0.37% and 18.18% of the total NTS collection [20]. In South India, the major source of NTS includes humans followed by animals and food products. In the West region of India, food products served as a major source of NTS while in the East region food products and animals contributed almost equally as a source of NTS. However, the major source of NTS in the North areas was animals. The prevalence of NTS in regions in India in descending order is as follows: South, North, West, East, and Central [46,13].

Emerging antimicrobial resistance

Natural or artificial compounds that may kill or prevent the development of microorganisms are known as antimicrobial agents. They frequently differentiate in terms of their mode of action, which can influence whether a certain antibiotic will be clinically effective in managing a disease [47]. Notably, some patients still contain Salmonella bacteria even after taking medicines that improve their symptoms. Due to the production of beta-lactamases, NTS strains are naturally resistant to penicillin, which can reduce the effectiveness of penicillin-based antibiotics in treating NTS infections. For instance, as a result of an infection recurrence, a course of antibiotics can lead to more positive stool cultures during the third week of treatment and more negative stool cultures observed in the first week of therapy [48]. Additionally, these antibiotics include a risk of adverse medication responses, such as rashes on the skin with ampicillin, leucopenia with cotrimoxazole, and urticaria, along with fluoroquinolones' ability to cause severe headaches, nausea, epigastric discomfort, and dizziness [49]. The possibility of tendinitis raises additional concerns about the use of quinolones in young children [50]. In most cases, people recover within a week or two, although some may experience more severe symptoms and require hospitalization. The infection has to be properly treated, and the development of antibiotic-resistant strains of the bacteria should be treated [51].

The epidemiology of non-typhoidal salmonellosis saw two significant modifications in the second half of the 20th century. First, populations of food animals have noticed an increase in Salmonella typhimurium strains that are resistant to several antibiotics. Second, Salmonella enteritidis has become a significant pathogen linked to eggs [49]. NTS produces mild, self-limiting acute enterocolitis [52]. The condition may also appear as a febrile-invasive disease, commonly without diarrhea, with localized infections that can be life-threatening [1,36].

Diagnosis and treatment 

Diagnosing NTS infections typically involves laboratory tests to confirm the presence of the bacteria. The most common diagnostic method is a stool culture, where a sample of stool is analyzed for the presence of salmonella bacteria [53]. The symptoms of NTS infections are similar to those of other gastrointestinal infections, making it challenging to distinguish NTS from other pathogens without laboratory testing. Moreover, obtaining reliable samples for testing can be difficult because the bacteria may only be present intermittently in the stool. Stool culture accuracy is impacted by sample collection, handling, and transportation. False-negative results may occur if antibiotics were taken before the test. Bacterial concentration can vary, and the infection may not be detectable at times. Multiple samples may be needed to increase the chances of detecting the infection. Culture testing takes 48 to 72 hours for bacteria to grow, delaying diagnosis and treatment. Blood tests may also be conducted if there is associated bacteremia or extra-intestinal infection. Urine or bone marrow samples may be collected for testing, depending on the presentation of the infection [54]. It has been suggested that the detection of Immunoglobulin G (IgG) to the Virulance (Vi) antigen be used to identify chronic carriers. The test has been beneficial when used in combination with epidemic research [55-58]. Its function in identifying carriers among people of all ages in areas of an epidemic where background levels of IgG to Vi may be high or where the Vi vaccination is often administered is less apparent [59]. Treatment of NTS infections often involves supportive care and management of symptoms [48]. The infection resolves on its own within a week or two without specific treatment. Hydration is crucial to prevent dehydration caused by diarrhea or vomiting. Rest and a balanced diet are recommended to support recovery. Antibiotics may be administered in specific circumstances, such as severe or persistent infection [60].

Antibiotics are often only used in high-risk patients or in cases when the infection has advanced beyond the gastrointestinal tract. Susceptibility testing should be used to direct antibiotic therapy to ensure efficacy [61]. If the diagnosis is confirmed and the strain is susceptible taking an oral fluoroquinolone for seven to 10 days at the maximal recommended dose, like ciprofloxacin. Oral azithromycin, also administered at the maximal dose for seven days, is a successful alternative if the strain is intermediately susceptible or resistant to ciprofloxacin. There are very few options available for dealing with a multidrug-resistant strain that is extended-spectrum beta-lactamase (ESBL) positive and resistant to ciprofloxacin and azithromycin, although they may include costly medicines like carbapenems or tigecycline. It is essential to finish the entire course of antibiotics to guarantee the eradication of the bacteria and prevent the development of antibiotic resistance. Hospitalization and intravenous antibiotics may be required in cases of NTS bacteremia or invasive infection. Treatment plans should be devised individually, taking into account the extent of the infection, the patient's age and relative condition, and regional trends of antibiotic resistance [62]. To detect salmonellosis, salmonella bacteria are isolated from stool, blood, or - less frequently - urine [63-65]. Salmonella bacteria can also be isolated using bone marrow aspirates. Bone marrow contains up to 10 times more bacteria than peripheral blood. When the bacteria are cultivated, they can no longer be present in the blood, after receiving antibiotic medication. But it might continue to exist in the bone marrow. Treatment typically involves antibiotics, hydration, and rest [54].

Prevention 

NTS infections in India require a multi-faceted approach. One crucial step is ensuring that food and water sources are properly treated and handled, which involves properly washing fruits and vegetables, cooking meat and eggs to the optimum temperature, and avoiding dairy items that have not been pasteurized. Controlling NTS encompasses assuring food safety from farm to fork in developed countries and improving sanitation infrastructure that comprises promoting greater cleanliness standards in public areas and enhancing sewage treatment and waste management systems [52]. Antibiotic therapy also does not eliminate NTS persistence and may lengthen the time that NTS is shed [66]. Campaigns to raise public awareness and educational campaigns in schools and community centers along with the use of social media to reach a wider range of audience are also crucial for fostering good hygiene and food safety habits [52]. Furthermore, it is essential to implement strict regulations and enforcement measures to ensure compliance with hygiene standards. This includes regular inspections of food establishments and imposing penalties on those found to be violating the guidelines. Additionally, collaboration between government agencies, healthcare professionals, and the public is necessary to effectively address the issue of NTS transmission and reduce its prevalence. By working together, we can create a safer and healthier environment for everyone.

Control

To decrease the nation's rising NTS infection problem, the Indian government has taken several actions [20]. In addition to investing in better sanitary facilities in rural regions, the Ministry of Health and Family Welfare has initiated several public awareness initiatives to encourage good food safety and hygiene practices [67]. The government has also put up a surveillance system to track outbreaks of NTS infections and act swiftly to stop the spread of the bacteria [68]. To minimize and handle NTS infections in India, several actions like reinforcing laws governing the food and beverage business, supporting better hygienic standards in public locations, and investing in better sanitation infrastructure in rural areas are imperative [69]. These measures will not only prevent the occurrence of NTS infections but also ensure a healthier population overall. Additionally, it is crucial to educate the public about the importance of proper food handling, cooking temperatures, and personal hygiene. By promoting these practices, individuals can protect themselves and others from the risks associated with NTS infections. Ultimately, a comprehensive approach involving government intervention, public awareness, and improved infrastructure will be key in effectively minimizing and handling NTS infections in India.

## Conclusions

In India, NTS infections pose a significant threat to public health, often overlooked due to inadequate surveillance. NTS mainly spreads by the fecal-oral route, consuming contaminated animal products, and using polluted water and fertilizers in agriculture, exacerbated by poor sanitation practices. Antimicrobial resistance is a growing concern and emphasizes the need for cautious antibiotic use. Prevention strategies include proper handling of food and water, improved sanitation, public awareness, and government initiatives supported by surveillance systems. Future control efforts should place a priority on regulatory strengthening, health promotion, investment in sanitation infrastructure, and dealing with health-related problems to decrease NTS infections effectively. 
